# Organizing pneumonia co‐existing with carcinoid tumour: complete resolution with bronchoscopic tumour resection

**DOI:** 10.1002/rcr2.409

**Published:** 2019-03-07

**Authors:** Divyansh Bajaj, Nevins Todd, Rydhwana Hossain, Karan Mahajan, Whitney Burrows, Ashutosh Sachdeva

**Affiliations:** ^1^ Medicine, St. Vincent's Medical Center/Quinnipiac University Bridgeport CT USA; ^2^ Pulmonary and Critical Care University of Maryland School of Medicine Baltimore MD USA; ^3^ Radiology University of Maryland School of Medicine Baltimore MD USA; ^4^ Medicine Presbyterian Rust Medical Center Rio Rancho NM USA; ^5^ Thoracic Surgery University of Maryland School of Medicine Baltimore MD USA

**Keywords:** Carcinoid tumour, organizing pneumonia

## Abstract

Organizing pneumonia is a well‐known clinical entity resulting in response to noxious stimuli causing lung injury. It is known to occur with infectious disease processes, neoplasms, post lung surgery or radiation therapy and when idiopathic, is called cryptogenic organizing pneumonia. We present an unusual case of a 48‐year‐old woman who presented with chronic cough and progressive dyspnoea while being on macrolide therapy for Lyme disease. Computerized tomography of chest demonstrated a well‐circumscribed nodule in the lingula and bilateral central ground glass opacities. Transbronchial biopsies were consistent with carcinoid tumour in the lingula and organizing pneumonia in bilateral lung fields. Bronchoscopic relief of obstruction was performed by mechanical debulking of the tumour, with subsequent complete resolution of bilateral opacities, consistent with resolution of organizing pneumonia without the need for steroid therapy.

## Introduction

Organizing pneumonia (OP), previously known as bronchiolitis obliterans, is a well‐known clinical entity. It is characterized by the inflammation of distal small airways and alveoli in response to infectious or non‐infectious stimuli. The exact pathogenesis of OP is not known; however, the disease has been seen in association with several connective tissue disorders, infections, haematological and solid malignancies, radiation, and chemotherapy. It has also been reported to occur due to lung tumours causing post obstructive lung injury, usually distal to obstruction. We report an unusual case of OP occurring as a consequence of obstruction due to a carcinoid tumour and its complete resolution by bronchoscopic relief of obstruction without the need for steroid therapy.

## Case Report

**Figure 1:**
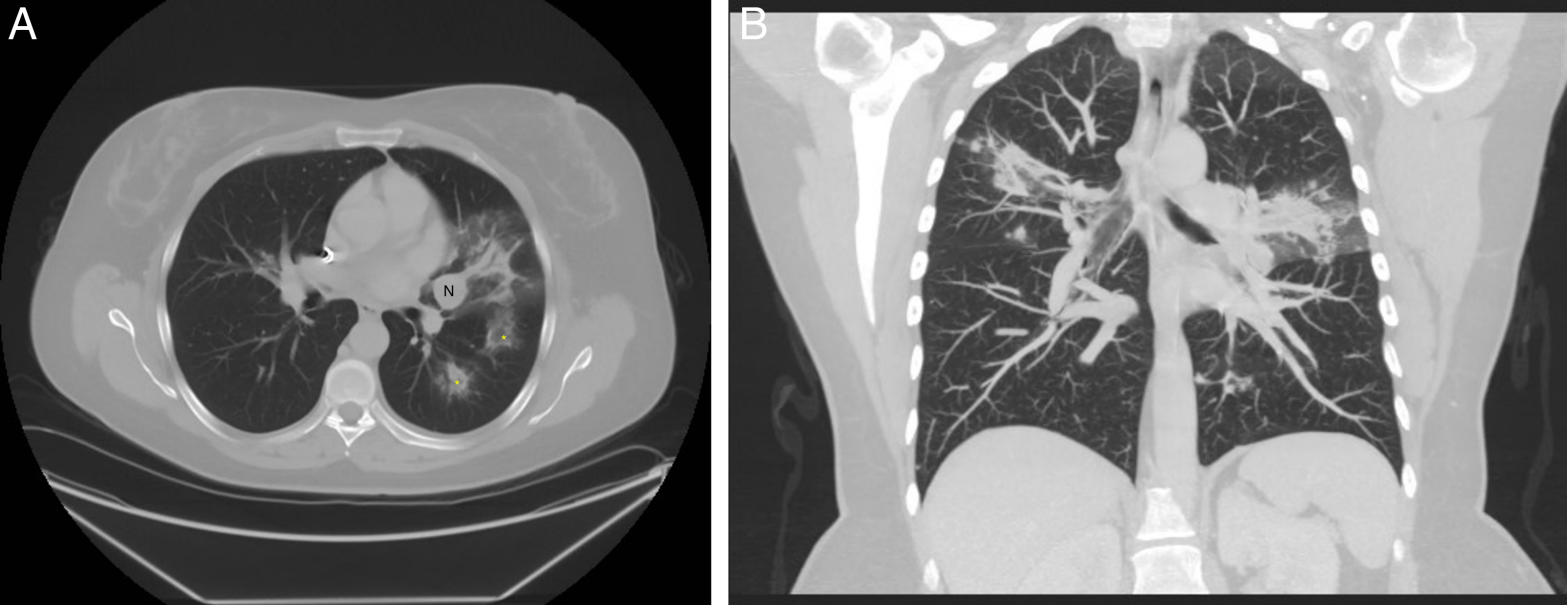
Pre‐bronchoscopy imaging. A Axial computerized tomography image demonstrating lingular nodule (labelled N) and surrounding lung opacities with reversed “halo sign” (asterisks) suggestive of organizing pneumonia. B Coronal image showing bilateral lung opacities and lingular nodule.

**Figure 2:**
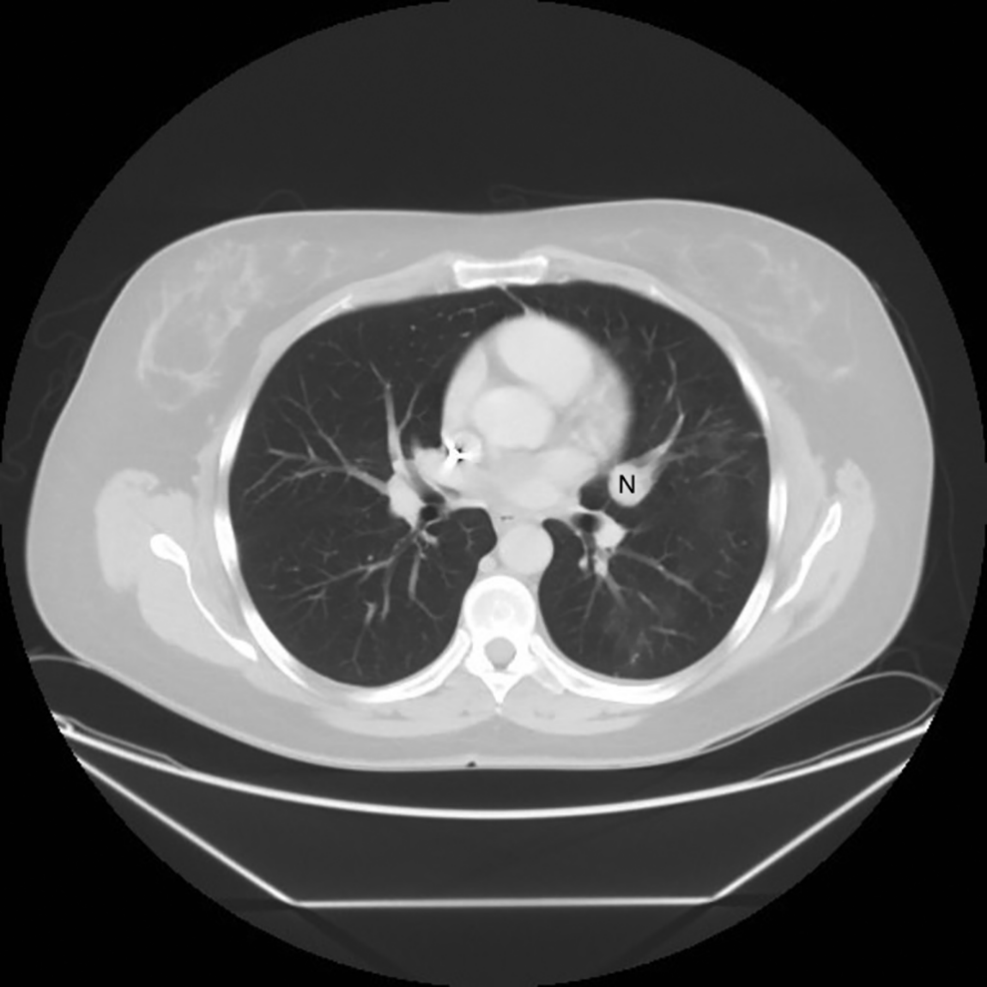
One‐month post‐bronchoscopy axial computerized imaging showing lung nodule (labelled N) near complete resolution of the lung opacities.

A 48‐year‐old woman, never smoker, with past history of Lyme disease presented with non‐resolving cough of six‐month duration and progressive dyspnoea on exertion. She was on chronic therapy with azithromycin, minocycline, and plaquenil for Lyme disease. Her prior workup included autoimmune serologies for connective tissue disease, which were negative. A contrast‐enhanced computerized tomography (CT) scan of the chest revealed a well circumscribed 16‐mm lingular nodule. Pulmonary functions tests revealed normal expiratory flows and lung capacity. A follow‐up CT scan of the chest was performed that revealed increase in the size of lingular nodule and associated bilateral central lung opacities. Some of the opacities demonstrated a central ground‐glass opacity surrounded by denser air‐space consolidation consistent with the reversed halo sign (Fig. 1A, B). Patient subsequently underwent diagnostic and therapeutic bronchoscopy and was found to have partially obstructing lesion, with intrinsic and extrinsic component, in the inferior lingula sub‐segment of the left upper lobe. Transbronchial biopsies of the tumour and lung parenchyma were performed, which showed carcinoid tumour and OP, respectively. Mechanical debulking of endobronchial tumour was performed using large (2.8 mm) biopsy forceps along with balloon dilation and therapeutic aspiration. A repeat CT scan of the chest was performed a month later and prior to planned surgical resection; this revealed near complete resolution of lung opacities (Fig. 2). Subsequently, she underwent left thoracotomy with lingulectomy for complete resection of the tumour and currently has complete resolution of her initial presenting symptoms.

## Discussion

OP is a type of interstitial lung disease that affects the distal bronchioles, respiratory bronchioles, alveolar ducts and sacs. The clinical manifestations of OP include persistent dry cough, fever, dyspnoea, fatigue, weight loss, and rarely haemoptysis. The radiological features of OP are variable and include bilateral patchy opacities, nodules, and unilateral focal or lobar consolidations. Reversed halo sign (Atoll sign) on CT scan was previously considered pathognomonic for the diagnosis of OP; however, it has been now known to be associated with many other conditions such as pulmonary tuberculosis, sarcoidosis, and invasive pulmonary fungal infections as well. In some cases, on imaging OP may closely resemble lung mass, making it difficult to differentiate between the two solely based on radiological findings. Moreover, OP has also been known to co‐exist with lung neoplasm secondary to endobronchial obstruction and usually distal to the obstruction as a response to post‐obstructive pneumonitis. In a study published by Romero et al., 37% of the specimens studied had OP in close proximity of the resected lung neoplasms 1. The authors proposed that this finding could be due to development of OP in response to endobronchial obstruction by the lung mass. OP has also been reported to develop at a site distant to the topographical situation of the lung tumour in several reports. In a case published by Sanchez et al., OP developed in the contralateral lung of the patient with adenocarcinoma 2. Enomoto et al. also reported a case where OP was found in topographically distant lung regions 3. In the above cases, steroid therapy was given, which is the mainstay for the treatment of OP, and the lung tumour was resected.

The development of diffuse OP in a patient after unilateral radiation therapy has also been reported in the literature. An immunological process triggering bilateral lymphocytic alveolitis has been proposed as the likely underlying mechanism for bilateral disease development 4. In our case, carcinoid tumour was found in the left upper lobe lingula resulting in diffuse OP. Bronchoscopic relief of obstruction resulted in complete resolution of OP without the use of steroid therapy. The resolution of OP after the removal of the obstructing tumour, even from the non‐obstructed regions, suggests that post‐obstructive OP is a diffuse rather than localized phenomenon occurring as a result of possible immunological response to the lung injury caused by the tumour mass or obstruction due to the tumour. Through this case, we hypothesize that relief of tumour obstruction or surgical resection may result in resolution of OP without steroid therapy.

In conclusion, clinicians should keep in mind that although rare, OP co‐exists with pulmonary neoplasms and can present as either a diffuse or localized opacities of the lung parenchyma. Our case suggests that steroid therapy may not always be required for the management of OP and complete resolution could occur with bronchoscopic resection of the tumour mass and relief of airway obstruction or surgical resection of tumour.

### Disclosure statement

Appropriate written informed consent was obtained for publication of this case report and accompanying images.
